# Opening up creative resources: towards age-friendly communities through rhizomatic thinking and doing

**DOI:** 10.1080/09650792.2024.2370277

**Published:** 2024-06-27

**Authors:** Marielle Schuurman, Barbara Groot, Tineke Abma

**Affiliations:** a Department of Public Health, Leiden University Medical Center, Leiden, The Netherlands; b Vormvinder, Eemklooster, Amersfoort, The Netherlands; c Health Sciences Department, VU Amsterdam, Amsterdam, The Netherlands; d Leyden Academy on Vitality and Ageing, Leiden, The Netherlands

**Keywords:** Age-friendly community, horizontal epistemology, participatory arts-based research, ecosystem-thinking, metaphors

## Abstract

Globally, many complex issues, like the ageing population and health inequalities, require attention. People are experimenting to combat these issues in their local contexts through bigger or smaller networks; however, much of the knowledge about these initiatives remains localised and elitist and omits the voices and perspectives of citizens. This article identifies the characteristics of a more horizontal, emergent and plural epistemology to mobilize knowledge. We used local networks building age-friendly communities in the Netherlands as a case study. With members of 110 local networks, we worked with a new methodology called *dynamic knowledge synthesis* to create fruitful interactions and learn with stakeholders, including older citizens, in a learning community. Four working principles helped us, namely (1) a rhizomatic design based on multiplicity, heterogeneity and non-linearity of knowledge; (2) fertile soil nurtured by the talents and wisdom of participants through participatory arts-based methods; (3) so-called ‘wicked skills’ of a forester 2.0 to facilitate learning; and (4) an ecosystem metaphor as a boundary object to understand local networks. We invite colleagues to experiment with dynamic knowledge synthesis to connect on different levels, with particular attention to the inclusion of citizens in creating sustainable local communities.

## Introduction

**Figure 1.**
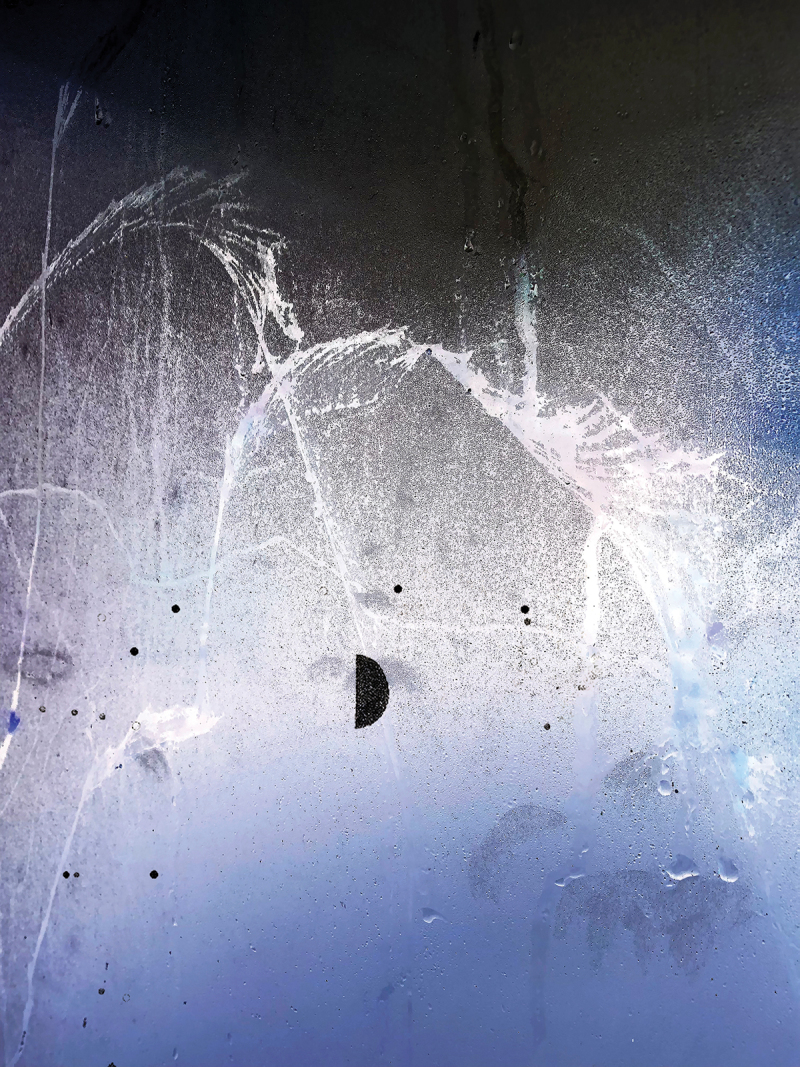
Not-knowing, artistic expression. Artistic expression made by first author.

Many complex issues, such as the ageing population, climate change, pandemics and health inequalities, are raising concern worldwide. For instance, COVID-19 showed us that wicked problems require analysis and actions that address structural political-economic conditions and ‘far less ordered, “unruly” processes reflecting complexity, uncertainty, contingency and context-specificity’ (Leach et al. 2021, 1). These unruly processes are often accompanied by emotions of confusion, fear and anger. People may wonder: ‘Is our future dark and full of losses or are there still beacons of light?’, ‘How do we navigate in an uncertain world where we can hardly see where we are heading?’, and, perhaps, ‘What happens when we capture these movements through images instead of language and rational thinking? (See Figure 1). Radically transformative, egalitarian and inclusive knowledge systems and initiatives are critical for the future, as they acknowledge affective, artistic and indigenous forms of knowing (Abma et al. 2019; ICPHR 2013; Wright and Kongats 2019). This aligns with grassroots emancipation and empowerment movements in policy, science and society, including responsible research and innovation practices (Abma and Groot 2023). From a research perspective, it calls for a transformation of our vision of knowledge production, fairer power balances between stakeholders, and research epistemologies and designs that embrace more egalitarian partnerships in which experiential knowledge is as welcome as other types of knowledge (Heron and Reason 2008). This is grounded in an awareness that various types of knowledge and perspectives can help to properly understand and address complex issues and reflect on actions (Bradbury 2015).

Although there are numerous initiatives and actors worldwide working on complex issues, many of these initiatives are still relatively small in scale, locally organized and not connected. Knowledge exchange between the initiators and members of these local transformative grassroots initiatives, national and regional officials, funding agencies, scientific and educational institutes, and other stakeholders is still scarce (Cook 2022). Instead, these local initiatives and networks tend to focus on their mission to transform their communities and make an impact locally rather than on knowledge generation or sharing beyond their locality. Knowledge exchange is often viewed only as a bonus in the process of learning with and from each other, but ‘learning in action’ (Dewey 1986) could be quite effective in gaining a more in-depth understanding of the process, the conditions and the pitfalls of these social movements and collective actions to transform our world into a more sustainable universe, as well as how inclusive knowledge and learning processes can be organized.

Many countries, governments, and universities are struggling with the major challenges facing our societies, and some have invested in different forms of participatory knowledge infrastructures over the last decades in which science, practice, and policy exchange insights and collaborate in actions to make an impact beyond academia. Examples of such collaborations include learning networks, academic collaborative centres, care networks, consortia or living labs (Oortwijn, Reijmerink, and Bussemaker 2023). While some of these are interdisciplinary, others are transdisciplinary in nature. Transdisciplinarity involves:

collaborative work between science and society, meaning that it involves access to other sources of knowledge outside of the strictly scientific sphere and therefore, is thought of as a more ambitious approach than interdisciplinary work.

(Morales and Muñoz 2021)

Transdisciplinary collaborations emerge when the problems at hand cannot be solved without collaboration with those who have access to sources of knowledge beyond the scientific (academic) domain (Morales and Muñoz 2021). However, university researchers and educators are often in the lead in these collaborations, and they have the power to make decisions regarding the research agenda, epistemologies, designs and methods used. A recent study on current participatory knowledge infrastructures has shown that collaborations with citizens and between different domains (e.g. cure and care) were fragile and disappeared when the project funding stopped (Oortwijn, Reijmerink, and Bussemaker 2023). Inclusiveness and trans-disciplinarity in these infrastructures are still lacking, as is the involvement of pioneers from local settings as meaningful partners.

The missing connection with grass-root pioneers and an inclusive, whole-system approach to participatory knowledge infrastructures is reflected in the challenges social movements encounter, such as the global age-friendly community movement (World Health Organization 2007), the focus in this article, because it offers a rich case to study newly developing transdisciplinary knowledge infrastructures. The age-friendly community movement encourages ‘active ageing by optimizing opportunities for health, participation and security in order to enhance quality of life as people age’ (WHO 2007, 1). The age-friendly community framework covers eight areas: outdoor spaces, transportation, housing, social participation, respect and social inclusion, civic participation and employment, communication and information, as well as community and health services. Transdisciplinary networks and approaches to policy, education, practice, community engagement and evaluation are necessary for this movement to realize a better quality of life for people of all ages, including older people (van Hoof and Marston 2021; van Hoof et al. 2021), but participation of older citizens is still meagre and mainly organized through consultation and steering committees (Novek and Menec 2014; Remillard-Boilard, Buffel, and Phillipson 2017). Initiatives on building age-friendly communities are rarely successful in allowing older people themselves to be central to the movement (Buffel 2015; Buffel and Phillipson 2024). These age-friendly community initiatives are also often not inclusive, due to ageism (Buffel et al. 2020) or lack of attention to marginalized groups such as older migrants and people from LHBTIQ2S+ communities (Lipinski, Stinchcombe, and Wilson 2023).

In this article, we present the main characteristics of a more horizontal, emergent and plural epistemology to mobilize knowledge for age-friendly communities. We use local networks that build age-friendly communities in the Netherlands as a case study. Alongside members of 110 local networks, we worked with a new methodology called *dynamic knowledge synthesis* to create fruitful interactions and learn with stakeholders, including older citizens, in a learning community.

## Methodology

Together
All different places
The variety of knowledge
The complexity of the topic
How?
Silence
Not-knowing
Just do
Reflect
Do
Why?
Silence
Reflect
Wow.

‘Elf’ poem about our methodology, written by the second authors in an online working session.

### Dynamic knowledge synthesis

One of the aims in our study was to develop a new research design called dynamic knowledge synthesis. The commissioning organization (ZonMw, the Dutch Council for Health Research) invited us to stimulate the learning process within and between 110 local networks for the care and well-being of older people, to bring in knowledge from outside these networks and to aggregate and synthesize knowledge from these bottom-up initiatives. The 110 networks focus on various topics, ranging from fall prevention to social meeting places. ZonMw financed all the networks for a certain period (one to three years). This research, also funded by ZonMw, had a dynamic and action-oriented character. In this study, we gathered, enriched and shared knowledge in several cycles with project leaders, action researchers, network partners and seniors of the local networks in order to explore, share and use insights about good living with and for older people in the community and to apply actions based on these insights in the community.

Our research design was grounded in a transformative paradigm (Mertens 1999). Those working with this paradigm incorporate a social justice orientation and advocacy for marginalized community voices. In this approach, researchers seek to include voices and perspectives that are often hidden or silenced in the search for epistemic justice (Fricker 2007). They attend to asymmetric power relationships and social inequality. In line with this paradigm, our study is inspired by principles of the participatory research design in the domain of health and social well-being in which researchers strive for social justice by collaborating with those who work and live in the subject of the study (Abma et al. 2017, 2019Wright and Kongats 2019). We worked with people building age-friendly communities in the Netherlands, including older citizens, professionals, project leaders and action researchers of 110 local networks nationwide.

### Research team and creative approach

The research team consisted of a participatory action researcher who is also a visual designer (first author), a senior researcher experienced in participatory arts-based research and ethics (second author), and a professor specialized in the participation of older people within society as well as in knowledge production systems (last author). The researchers were guided by the experiences and daily practices of those involved and their need to learn about them. They had a vision of what Abma (2020a, 2020b) in line with Kunneman (2013) has coined a ‘horizontal epistemology’ in which all kinds of knowledge are relevant and necessary for epistemic justice (Fricker 2007).

### Methods of data generation and analysis

We used participatory arts-based methods (Seppälä, Sarantou, and Miettinen 2021), such as theatrical, musical and visual ways of working, to learn and reflect together and to gain a deeper understanding of experiences, including existential knowledge that is often unsayable or hard to explicate (Visse, Hansen, and Leget 2019). We also worked with metaphors (Lakoff 1993; Northcote and Fetherston 2006), because we noticed the high energy level of the people involved when we deliberately used certain metaphors that participants brought in themselves. This approach created space for different types of knowledge: academic, professional, experiential and artistic knowledge (Heron and Reason 2008; Abma et al. 2017).

This article is based on desk research of the accountability reports from the networks to the funder (*n* = 79 networks), twelve online creative meetings (*n* = 34 stakeholders from the networks), four physical knowledge workshops (*n* = 15 project leaders and seniors), three meeting days with a creative approach (*n* = 170 stakeholders from the networks), two sounding board group meetings with creative exercises (*n* = 8 staff of the funding organization) and individual interviews with stakeholders in the networks (*n* = 25). Moreover, we also wrote two Dutch articles with partners from the field (Schuurman et al. 2023, 2024) and created various graphic images, including a visual tool for collaborative learning about local networks (Schuurman, Groot, and Abma, 2024) and a small collection of zines (Piepmeier 2008; Triggs 2010). Zines – short for fanzine or magazine – are small-circulation self-published works of original or appropriated texts and images, usually reproduced via a copy machine and aimed at a minority audience.

Between the moments of creating, generating and unfolding knowledge, we facilitated moments of individual and collaborative analysis and synthesis. This synthesis was partly traditional and partly more artistic and unconventional. During the data generation sessions, there were moments of collaborative analysis through creative writing exercises. In smaller analysis sessions, we made zines to synthesize knowledge from different sessions. Using the steps of Collaborative Creative Hermeneutic Analysis (CCHA) (Lieshout and Cardiff 2011), we made collages in a small magazine format to find and depict the more unsayable themes with a visual language. These zines were also converted to small videos by a multimedia designer to facilitate sharing with a wider group of stakeholders. This conversion step was an extra layer of analysis and validation of the insights by stakeholders’ comments on the concept versions of the videos.

Reflexive thematic analysis (Braun et al. 2023) was used for the desk research and to analyse the transcripts of most sessions. Finally, the first author used artistic ways to access the more unsayable forms of knowledge (Balkema and Slager 2004; Borgdorff 2010) to reflect on topics and themes (e.g. Figure 1). This resulted in a visualization of knowing and not-knowing as both light and dark, clear and hazy, strange and familiar simultaneously. As we often encountered this doubleness in this study, we will provide a deeper explanation in the paragraphs below, especially in the section on rhizomatic design.

### Quality and ethical considerations

In this study, we used the quality criteria of the ICPHR (2022a), which are (1) participatory; (2) locally situated; (3) a collective research process; (4) collectively owned; (5) aims for transformation through human agency; (6) promotes critical reflexivity; (7) produces knowledge which is local, collective, co-created, conversational and diverse; (8) strives for a broad impact; (9) produces local evidence based on broad understandings of generalizability; (10) follows specific validity criteria; and (11) is a dialectical process characterized by messiness. The validity criteria we followed were participatory, intersubjective, contextual, catalytic, ethical and empathic validity.

The Institutional Review Board of the Medical Ethical Committee Leiden-Den Haag-Delft approved the research protocol (nr. 22–3072). In this protocol, we followed the ethical guidance of the ICPHR (2022b). Important aspects were: ‘respect for research participants’ – enabling participants or their representatives to make considered choices about whether and how to engage, as well as treating them respectfully throughout the research process, and ‘justice and fair treatment’ – ensuring that the costs and benefits of the research were distributed fairly, including enabling research participants to access the benefits of the research. All our knowledge sources have been published openly on the project website: www.agefriendlycommunity.org.

## Results

In the results, we present the highlights of our journey during the dynamic knowledge synthesis we conducted about age-friendly communities. We use the metaphor of an ecosystem to present our research approach because it aligns not only with the way stakeholders ideally built age-friendly communities but also with the methodological approach in which we worked with a diversity of knowledge, creative data generation and data analysis to put into practice our vision of horizontal epistemology and aim for epistemic justice. Below, we present the key lessons drawn from our study.

### Rhizomatic design

#### A participatory knowledge infrastructure based on horizontal epistemology

We approached the knowledge of local networks in this study as part of a rhizomatic design: similar actions and movements emerged in various places and local conditions, all connected on a deeper level. In their seminal work, *A Thousand Plateaus*, Deleuze and Guattari (1988) introduced the rhizome as an alternative for the strongly anchored tree with one trunk and a crown of leaves. This metaphorical rhizome is comparable to a ginger root: it grows in a non-linear way and in diverse directions, and these directions are mutually connected and interrelated and together form a plateau. Another characteristic of the rhizome is the uncertain, unpredictable and unexpected outcomes, which are the result of a pattern of actions and reactions. These actions and reactions do not flow from a rational plan-making actor, but rather emerge as chains of responses mobilized by the interaction of actors and their environment. The rhizome offers opportunities to rethink our research strategies, which are traditionally shaped by notions of strong foundations and fixed order, building knowledge towards clear goals, and following linear and predictable pathways. These strategies do not properly address the complexities of the world in which we are living.

Rhizomatic thinking challenges participatory action researchers to acknowledge their embeddedness; to let themselves be guided by the research topic, the needs of the heterogenic group of participants; to open up to the unexpected, multiple, heterogenous and yet interconnected actions and movements; to seize occasions that offer chances for transformation; to acknowledge that attempts to synthesize knowledge are never finished; and to bring in knowledge from the margins (Amorim and Ryan 2005). The rhizomatic design sees knowledge as emerging horizontally and close to the ground instead of the vertical [tree] approach prevalent in scientific research in general (Abma 2020a, 2020b; Kunneman 2013). In the currently dominant theories of knowledge, the expert stands above the nonexpert, the objective is valued higher than the subjective and the rational stands above the emotional, as symbolized in the knowledge pyramid (Sackett 2000).

Participatory research with various people – in this study, older people, their families, volunteers and professionals in various domains – requires the recognition of epistemic plurality (multiple forms of knowledge) and knowledge systems that are more horizontal (coexistence of knowledge forms). In line with the rhizomatic approach, according to Abma, 2020a, 2020b) and Kunneman, 2013, 2017) a ‘horizontal epistemology’ values scientific knowledge for objectification (Mode 1 knowledge) but equally values practical-professional knowledge developed by practitioners in their practical work with older people to solve problems (Mode 2 knowledge), as well as older people’s experiential knowledge about what it is like to grow older in their specific context, including existential issues like loneliness, transience and finitude. It also values the moral issues that are part of professional practice (Mode 3 knowledge).

In line with the rhizomatic design, we created a collaborative learning process with project leaders, professionals, action researchers and older people. We jointly determined the themes that people wanted to learn about together. We then facilitated multiple (online or offline) working sessions on these themes, in which we used arts-based methods to exchange knowledge and experiences about the implementation and impact of local networks and collaboration with older people. The knowledge we gained during these sessions was three-layered: We learned about age-friendly communities [content], transdisciplinary collaboration [process] and dynamic knowledge synthesis [methodology].

##### Example: drawing session – “the knowledge in my backpack”

**Figure 2.**
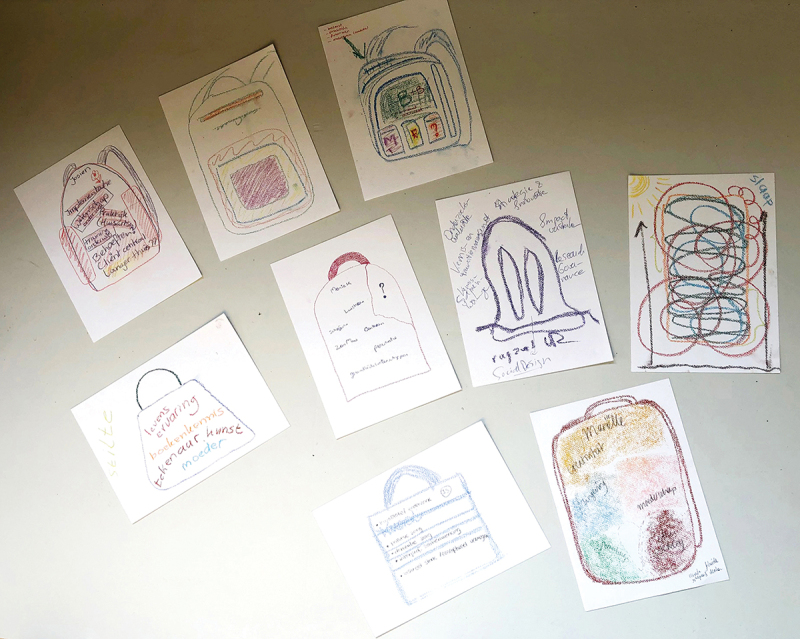
Drawings of backpacks with knowledge, made by officers of the funding body.

In a first low-profile drawing session, we examined our relationship to knowledge with funding agency members. In Dutch, there is a saying that everybody has a backpack filled with lived experience. So, we asked, ‘What knowledge do you have in your backpack?’ All the participants (implementation specialists, programme managers, directors of funding programmes) drew a (hand)bag or backpack of all their knowledge (See Figure 2). A profound dialogue then followed about our perspectives on knowledge and the discovery of what we called ‘hidden’ knowledge, or ‘potential’ knowledge and the importance of space for not-knowing.

My backpack contains a large part of motherhood, but also knowledge about nutrition and cooking. This knowledge seems to be separate from my professional knowledge: all the different subjects are separated from each other in different pockets.

Behind this zipper there is a very deep compartment, containing all the books and articles that I ever want to read, everything within me that I want to develop further but that I don’t get around to. […] This zipper also has to do with my identity. Who am I, other than a mother?

Somewhere behind the zipper lies the knowledge that belongs to us but is not part of our professional identity. Imagine what would happen when we open up that zipper and use all the knowledge and experience that we have gathered during our lives. The drawing and collaborative sense-making of these images appealed to the imagination and Mode 3 knowledge that arose in the conversations. Through this creative method, participants also gained insight into the multi-layered nature of knowledge and different types of pieces of knowledge. This contradicted their traditional vision of (expert-driven) Mode 1 and Mode 2 knowledge.

##### Example: Tableau vivant

In different working sessions, we used the method of constellations (Baur, Breed, and Visse 2022; Scholtens et al. 2021) through which all participants gained in-depth insights into mutual relationships and friction in cooperation in the neighbourhood. This was a collaborative activity with older people and others relevant to the local networks. The following is an example of a story about constellations described by one of the participants:

We were divided into groups and asked to portray, through tableaux vivant, a situation where relationships between those who need or receive care and informal and formal care are visualized.

We were lucky with our group: people were enthusiastic, and roles were almost automatically divided. We have a great ‘older adult’ and even our photographer who ensures we can self-reflect during this short exercise (See Figure 3a). We do this with humour, which boosts our cooperation. While laughing, we move on.

**Figure 3a.**
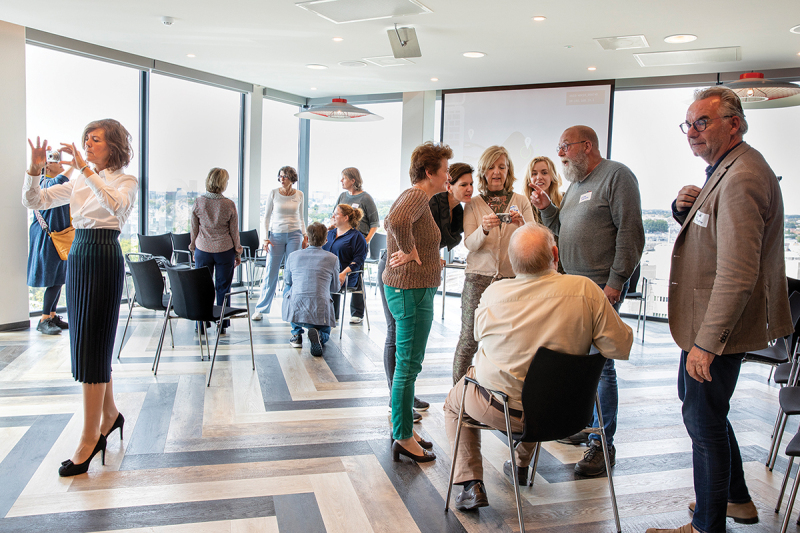
Impression I. An impression of an exercise with Tableau Vivant.

We talk briefly about the relationships that often arise between older people, their relatives and care professionals and relatively quickly conclude that one picture would not do justice to this complexity. We decided to create a series of placeholders to map the dynamics of the relationships between various actors.

First, we wanted to show that the older person is surrounded by love and care within their family/environment (See Figure 3b). You can see that some offer care, take other paternalistic stances and tell the person what to do. The older person himself does exactly what the environment expects of him: he suffers. He is in pain and is not a conversation partner but a recipient of care.

**Figure 3b.**
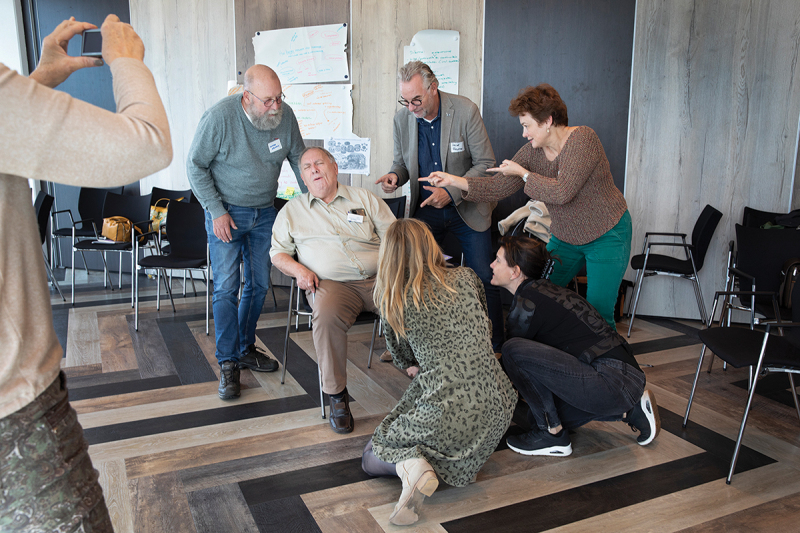
Impression II. An impression of an exercise with Tableau Vivant.

This was followed by the next picture (See Figure 3c). Here, you see informal care moving into formal care, demanding that the caregiver do something to alleviate their elder’s suffering. The member of our team, digressing in the back centre, is a representative of formal care who cannot cope with this pressure and demand.

**Figure 3c.**
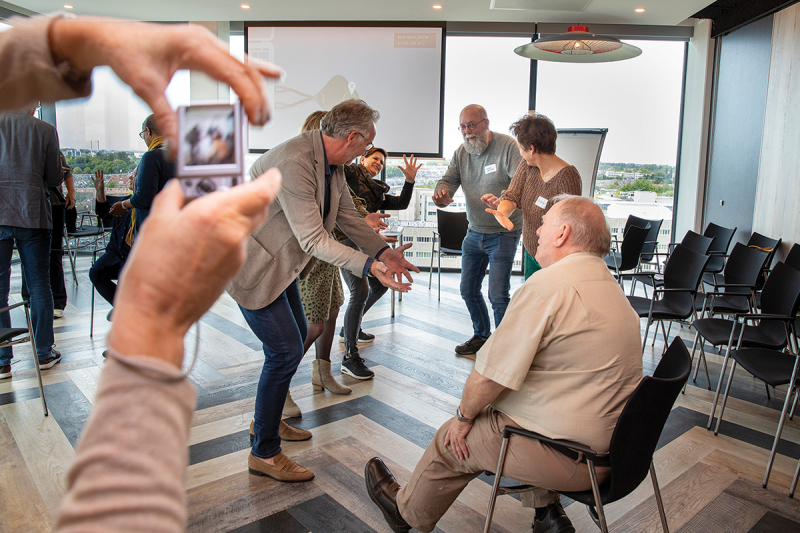
Impression III. An impression of an exercise with Tableau Vivant.

Finally, the final picture followed (See Figure 3d). It shows a complete attrition of all parties, each struggling with their own problems. Some are still writing prescriptions for drugs; others are just dead tired. They are still not talking to each other, nor to the older person. They are struggling with their own problems and seem distraught. The older person falls outside their circle.

**Figure 3d.**
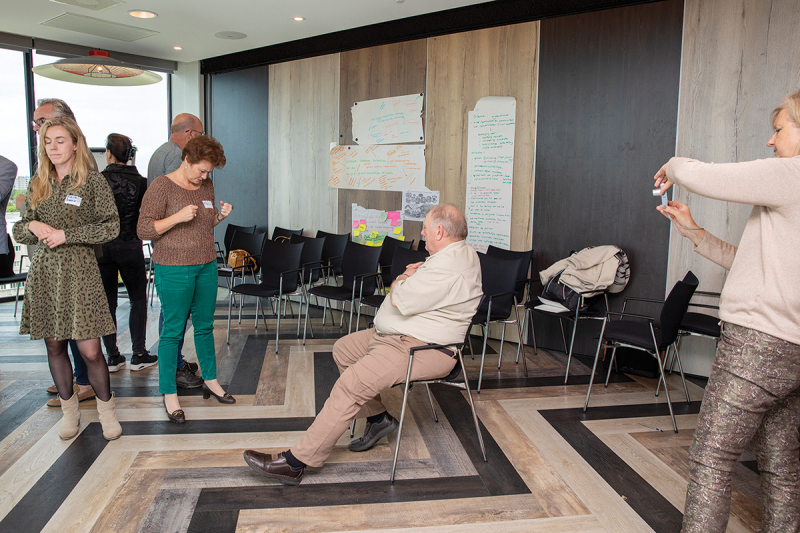
Impression IV. An impression of an exercise with Tableau Vivant.

This example shows how the lived and embodied experiences of all those involved were critically and quickly analysed. Embodied experiences, as well as their relation to each other, provided new forms of knowledge, especially Mode 3 knowledge. This form of knowledge is moral and relational about the mutual dynamics that often remain unsaid because they are also uncomfortable. Working with Mode 3 knowledge helped us to connect as a community on a much deeper level, and made people realize that knowledge is often difficult to express in words.

### In fertile soil

#### Using creative data generation to invite potential knowledge and not-knowing

During this study, we saw outstanding commitment and motivation among participants to learn together and contribute to age-friendly communities, as mentioned in the examples above. We used creative methods instead of more traditional data generation methods, such as questionnaires and interviews, to connect as much as possible to participants’ experiences and invite them to contribute to a dialogue. In online sessions, we combined creative writing and drawing. In live sessions, we provided tactile and natural materials to draw and build with, as well as working forms that stimulated embodied knowledge, such as the constellations method mentioned before. This created a warm and safe environment where participants were invited to share their experiences and co-create new perspectives. It was important for creating fertile ground in which something new could emerge, such as the metaphor we started working with:

##### Example: an online knowledge workspace about sustaining your local network

With a group of 18 people from local networks (project leaders, action researchers, older citizens and welfare professionals) in an online knowledge workshop, we shared experiences about sustaining the network after the subsidy period, which is usually a challenging phase for most network members. People were struggling in their local context and longed for a new kind of language to reflect on this complex topic. Here, the designer introduced the option of finding a metaphor for this complexity, as metaphors create powerful insights, because they invite us to see similarities in seemingly different perspectives. This is why metaphors have long been used in settings where people learn as a means of opening the way for new understanding (Northcote and Fetherston 2006).

**Figure 4.**
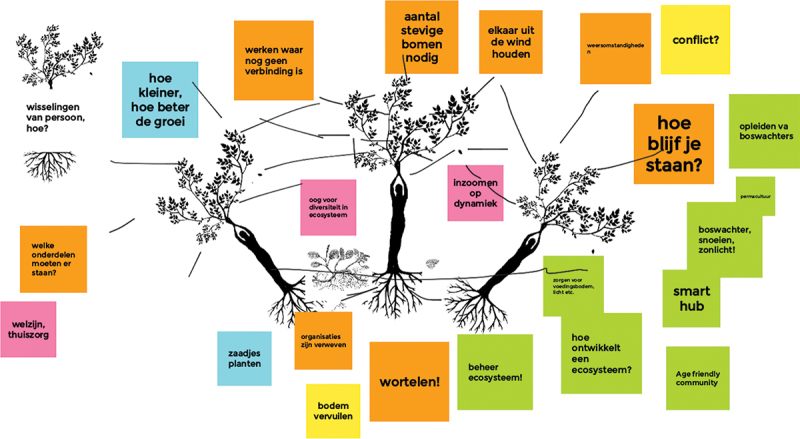
Exploration of the forest as a metaphor. A first joint exploration of the forest as a metaphor for healthy local networks.

**Figure 5.**
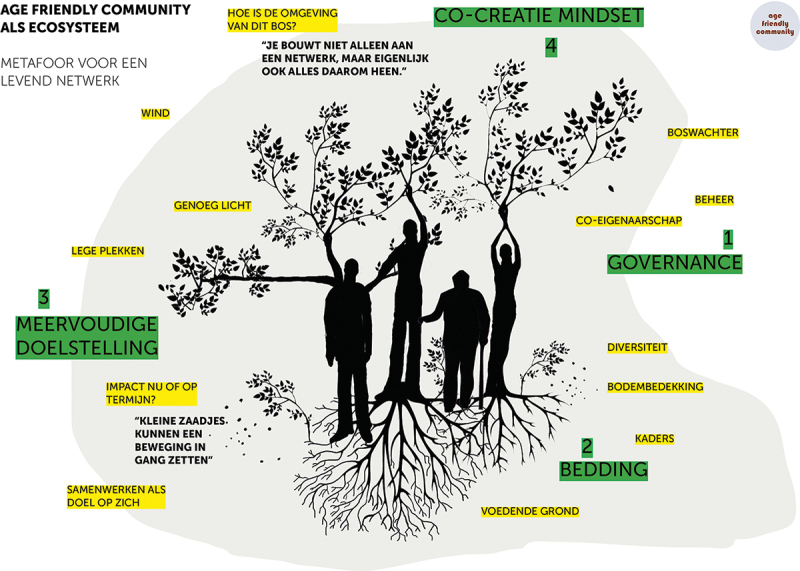
Second exploration of the forest as a metaphor. A second joint exploration of the forest as a metaphor for healthy local networks.

**Figure 6.**
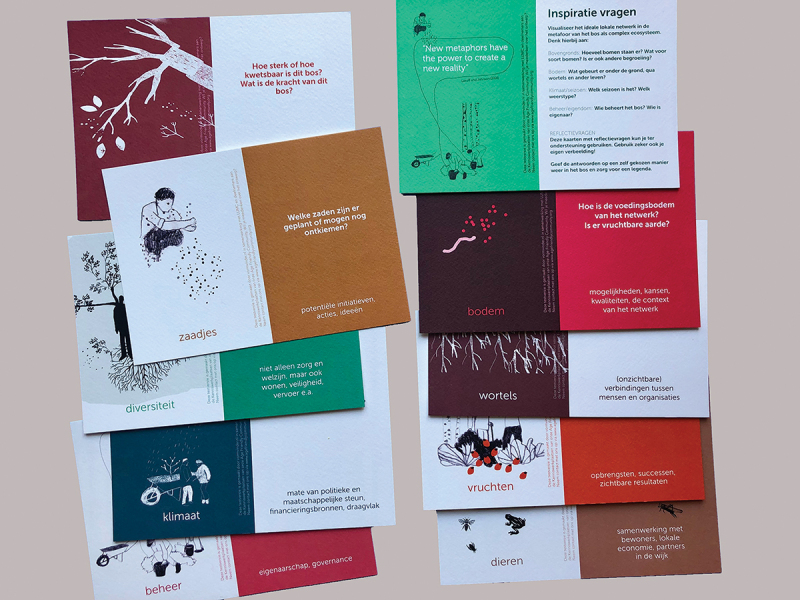
Working Together like a Forest, Tool for Local Network. Tool, co-created with stakeholders, to work with the metaphor in local settings (Schuurman, Groot, and Abma 2024).

To ease this process of finding a metaphor, the designer presented images of words already used in people’s language when discussing the subject: seeds, messiness, mud, spiderwebs, trees, roots, flowers and fruits. By combining a selection of images and connecting them through elementary drawing, we made a visual metaphor with the whole group: a complex ecosystem with trees, entangled roots, flowering branches, biodiversity, seeds and humus-like ground. All was connected and depended on the surrounding climate. This metaphor – created together in only a few minutes – lifted the discussion to a higher level. It helped to clarify difficulties around sustainability and vital communities on a more general level. Speaking of an ecosystem with all sorts of interconnected elements, the group found a language to share the complexity of their experiences and simultaneously perceive possible solutions. The visual metaphor helped to link the abstract implementation theme to personal experience and to tie imagination to analytic thinking. This metaphor also connected our efforts in local networks to a more prominent theme: the urgency of working towards a sustainable future in co-creation with each other and our planet (See Figure 4). After several workshops with different groups of people belonging to the network, as facilitators we synthesized the findings to provide feedback to all networks and fed back pieces of knowledge to the local settings (See Figure 5). Different people were thus given access to a variety of knowledge. Moreover, we co-developed a tool to work with the metaphor in local contexts, without the need for professional facilitators or a designer to join these sessions (See Figure 6).

##### Example: introducing the ecosystem metaphor to other stakeholders

Surprised by the power of the metaphor and the way it was embraced by the group as a new language, including by adapting it to their own local networks, we decided to share our experiences during the meeting with professionals from the funding agency. Although this felt vulnerable for us as researchers, we were supposed to deliver practical data about transdisciplinary networks and applicable solutions for building age-friendly communities (Mode 1 and Mode 2 knowledge). We showed some of the sketches we made with the group and talked about local networks as if they were forests. First, there was silence. Then, one of the board members said: ‘So if these local networks are forests, are we “state forest managers” and is it our role to govern these forests? How can we do this in a way that supports flourishing and diversity?’ A discussion followed about governance, strategy, responsibility, and sustainability. This produced a level of discussion and commitment we would never have reached with only words and our analytical brain. The metaphor of the forest as a complex ecosystem was open enough so people could connect to it in a way suitable for them. It also helped to create connections on a different level and invited analytical ways of thinking, imagination and creativity, which are necessary skills for solving wicked problems (Vandamme 2022).

### A new kind of forester with wicked skills

#### About facilitating the process

In this study, creating a participatory knowledge infrastructure with different stakeholders required researchers to act as facilitators creating communicative spaces to match the needs for learning and sharing of all involved. The concept of communicative space originates in the work of (Habermas 1987; in: Abma et al. 2019), who describes the ideal place for people to come together as a place of mutual recognition, change of perspective and willingness to empathize with and learn from the other. The examples above show that creative methods helped to foster a communicative space, with facilitators connecting people, types and pieces of knowledge and ideas. As Vandamme (2022) argues, facilitators of the future have so-called *wicked skills*. These competencies focus on a more holistic, systemic, relational worldview that embraces complexity. Wicked skills include paying attention to the multi-voiced identity of people and oneself, incorporating unconscious processes, working with paradoxes and ethics and being open to unexpected inspiration.

In this study, we noticed that these wicked skills were not only necessary to accommodate the participatory research process (Banks and Brydon-Miller 2018) but also to navigate critical questions and work with resistance. It was often difficult to stand for our research paradigm and approach in a traditional research context. In such a context, people have different ideas about the goodness of scientific research and are often not familiar with or educated in more emancipatory and transformative approaches to research.

##### Example: the canon and scientific community in a medical faculty

Our work is situated and embedded in a medical faculty and university hospital. This is a context where the adoption of participatory research approaches has been slower than in other domains (Abma et al. 2019). This relates to the vertical epistemology that is still dominant in the natural sciences and the value put on professional knowledge and expertise, particularly in highly specialized medical fields. The scientific commission of the medical faculty was therefore not used to assessing proposals like ours and found it difficult to review the open-ended proposal in which the goals, questions, methods, sample size and types of analysis were deliberately not set a priori to leave room for the stakeholders to engage and let the design emerge. As a result, the commission asked for a review with major revisions. For us, this posed a dilemma: Do we stand for our open-ended, more holistic and participatory approach, or do we follow the traditional approach to gain permission of the scientific commission? We reasoned the commission could only approve the proposal if we adopted their vocabulary to explain our plans, but without giving up on our values and ideals. So, for example, instead of preordaining the issues and concerns of our study, we focused on the actions, methods and stakeholders to be included. We emphasized the societal impact of our work as a major goal, but also gave an overview of the state-of-the art of the field, what we intended to add to this body of knowledge and what kind of quality and ethical criteria we use to legitimate our work. This is typically the boundary work that needs to be done to persuade and educate peers who are not familiar with participatory action research.

##### Example: our own resistance as people who are not educated about embracing not-knowing

Another issue related to being a new kind of researcher-forester was embracing the not-knowing for ourselves. We have been socialized to act as experts who know what to do and are not used to appreciating the not-knowing. How, then, could we create space for the not-knowing? How could we do this collaboratively with the participants in this study? The following example of the struggles of the facilitators in this study is taken from a collaborative diary:

One author: I am in the studio. We have conceived the plan to analyse the knowledge we have collected so far creatively and present it so that others can absorb it affectively. That others ‘feel’ the knowledge. Understand it on a deeper level. Ideally, we work on this together with those involved. However, we, ourselves still need to learn how to do this. We do not even know what we are doing.

**Figure 7.**
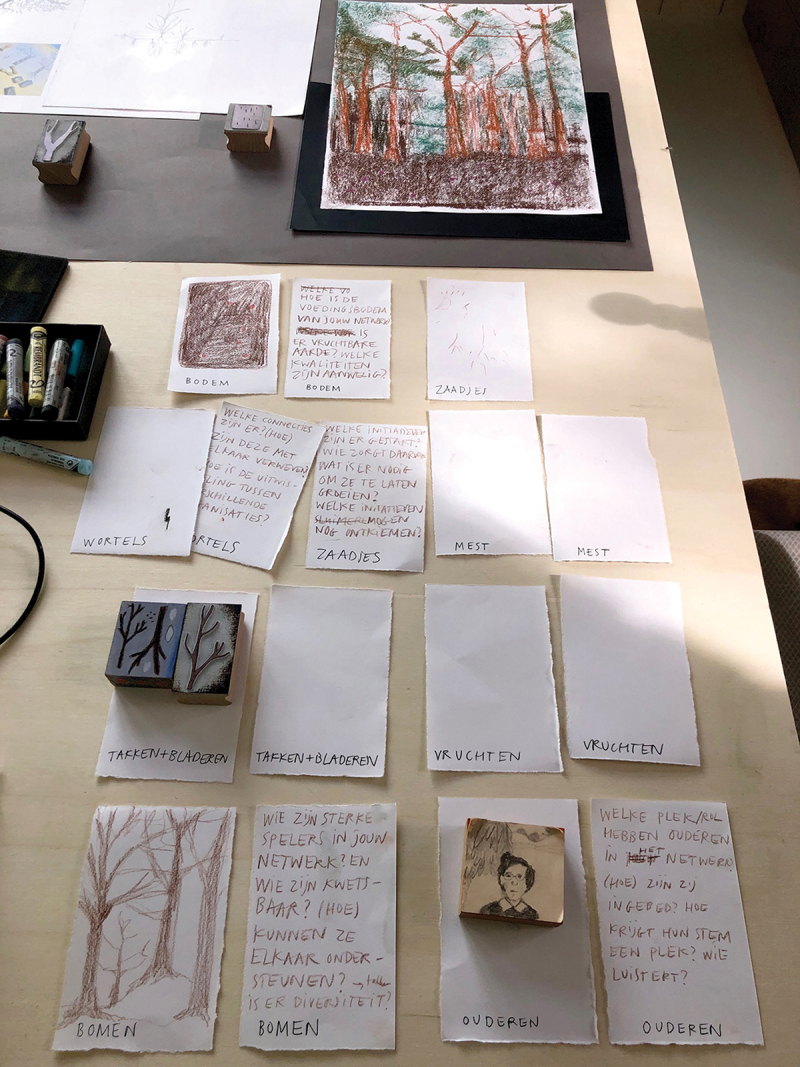
Zine-making. Zine-making process.

Mariëlle explains the working method: We will make a zine from one double-sided A3 paper, afterwards folded to A6, under pressure within half an hour. As an assignment beforehand, I had gone through the data on the subject I am making a zine about. I start. Tear out some texts. Some images. In a very short time, a story emerges in my head. I came up with themes I had not thought of before. A visual language for the story. One that expresses emotions (See Figure 7).

**Figure 8.**
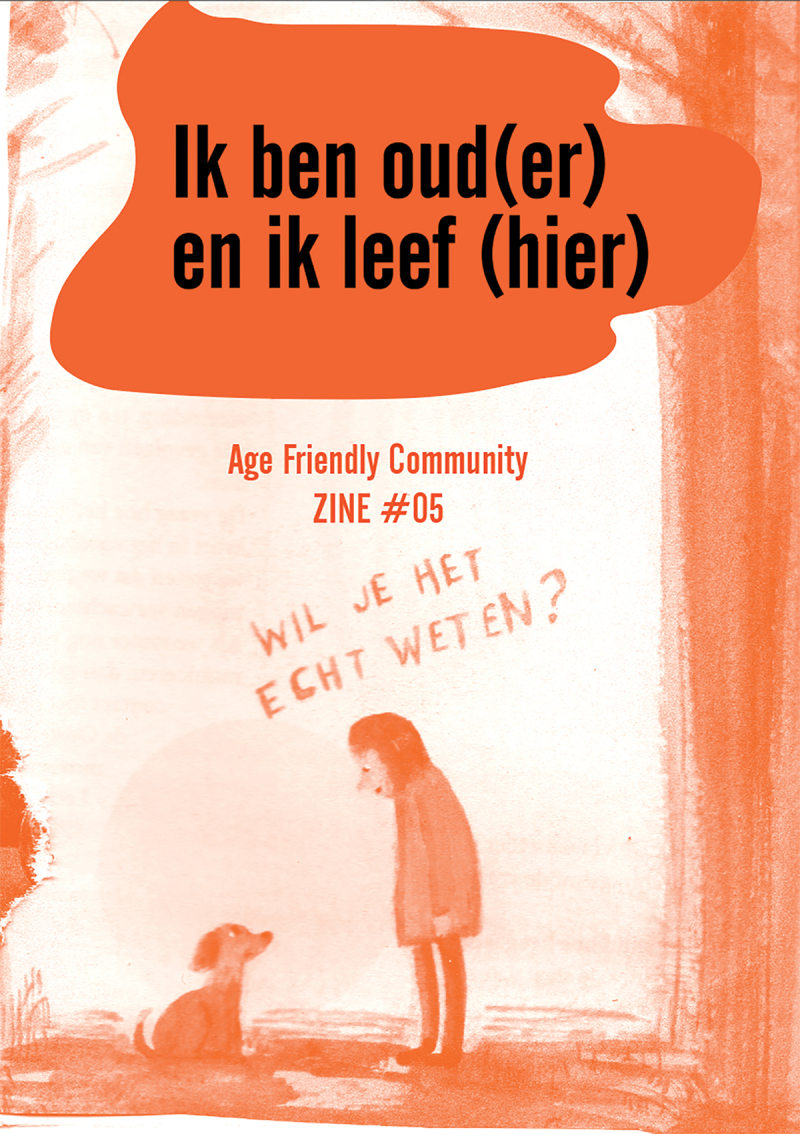
One of the zines developed in this study.

An example is this visual (See Figure 8). [The title in this figure in English is ‘I am (more) old, and I live (here)’. In the image the person says to the dog: ‘Do you really want to know?’]. It is typically what I felt when I heard the stories of older adults in the networks, but they did not say it in this way. However, this visual shows exactly my own affective feeling about the data.

The other author: Doing dynamic knowledge synthesis. But we don’t know what it is. Not-knowing is a significant factor in this process. We have never done it. The not-knowing, the doubt. How do we deal with that? Also, towards other stakeholders. Who expects what? How do we organize such a process? There is a tendency to seek Mode 1 or Mode 2 knowledge. Also, in us. Making products. Again and again. Within a deadline. But… we would like to make space for Mode 3 knowledge… i.e. more relational, moral, hard-to-put-into-words knowledge. We should make space for that. However, this also brings uncertainty and ambiguity. For us, as a facilitator of the research, as for all those involved. Others think you generate ‘objective knowledge’, so there is also resistance.

This example shows that not-knowing could be embraced, but this openness calls for skilled facilitators to slow down and embrace slow science in a highly dynamic field. In this study, we saw the value of silence for the quality of knowledge generation and analysis. It brought an openness to affective knowledge that is often overlooked. We believe such ‘openness to affect can negate preconceived conceptions of research that stagnate towards the norm’ (Clarke and Parsons 2013, 41).

### Ecosystem metaphor

#### A boundary object that connects on multiple levels

Finally, in this study, we saw again the value of a ‘boundary object’ (Leigh Star 2010; Star and Griesemer 1989) in participatory studies (Groot and Abma 2021). A boundary object is something concrete or abstract that provides as a starting point for dialogue with different stakeholders, perspectives and languages. In participatory studies, boundary objects are helpful in starting citizen engagement and collaborations of citizens with other stakeholders around a topic of interest (Groot and Abma 2021). Boundary objects are produced, using arts-based methods, to start a dialogue with stakeholders, aiming at connection and involvement in a mission towards transformation. Theory has shown that a successful boundary object evokes emotions among those who created the objects and those are encountering them (Amundsen and Hermansen 2021; de Kock et al. 2023; Green 2013; Groot and Abma 2021; Melo and Bishop 2020). The ecosystem metaphor we used in this study was a boundary object that was provocative and engaging. People felt drawn to use this metaphor to discuss and think about their network and the challenges they faced. It fostered change in how they regarded themselves, their own local environment and their agency on a more strategic, national and even global level.

##### Example: the metaphor as a boundary object, using nature-based materials

**Figure 9.**
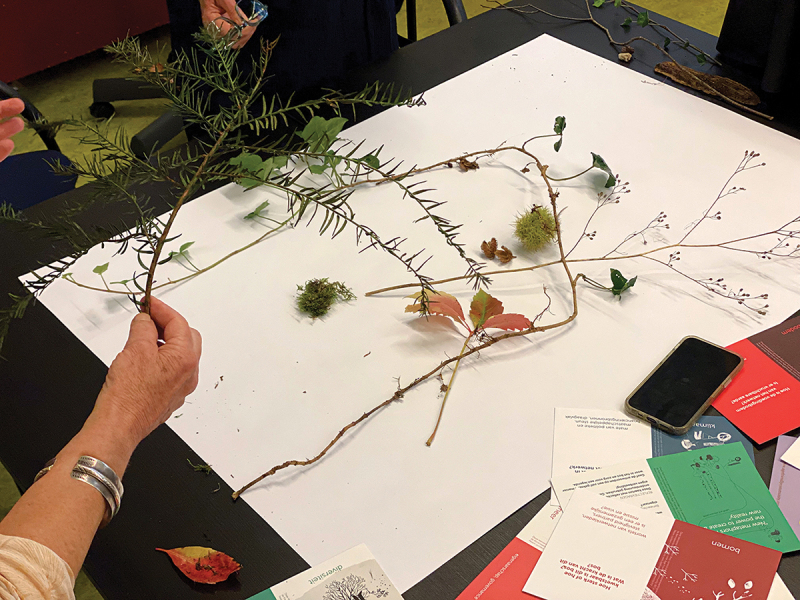
Working with the tool during a workshop with natural materials.

The following example shows how versatile the ecosystem metaphor is as a boundary object, and how it can provide unexpected knowledge and stories as well as Mode 3 knowledge. The conversation below was recorded in a national meeting with older people, project leaders and action researchers, people were invited – using the metaphor of the forest as a complex ecosystem – to look outside for materials (see Figure 9) to represent their own network:

Older adult 1 [colourful leaf in hand]: ‘Colourful. I just heard someone say: “It’s coming to an end”. But till its end, it remains colourful. Let’s keep that in mind especially.’

A project leader: ‘Transience … ’

Older adult 1: ‘No, not just impermanence, but colourful impermanence. We [older people] are very often framed as not colourful anymore. We are grey…’

Older adult 2: ‘True.’

Older adult 1: ‘So we keep that colourfulness, although we are ageing.’

Older adult 1: ‘You [project leader] did say decay as a negative. For me, it is very positive. The experience. The knowledge. That disappears into the ground [if you die], of course. But that, in turn, is food for others.’

Project leader 2: ‘If you include that in the network… If you start sharing that and keep sharing that…’

Project leader 1: ‘But the people in the network, we do not always see what they can do.’

Older adult 2: ‘True, some [shows a seed ball] do not have seed anymore. For others, it’s still in there. If you symbolize this as a living individual, then this one has had its time. But this one still has lots to bring. You can still “paw” that one.’

Older adult 1: ‘This metaphor brings out incredible things. A lot of roads out of the ecological forest. It’s about “decay”, which in turn feeds the soil and what has yet to come. So, what then, is decay? The red leaves are very much alive. While it does come to an end. And then that very long ivy, symbol for that long network, that goes all over the world, so not only in this forest, but also far away.’

**Figure 10.**
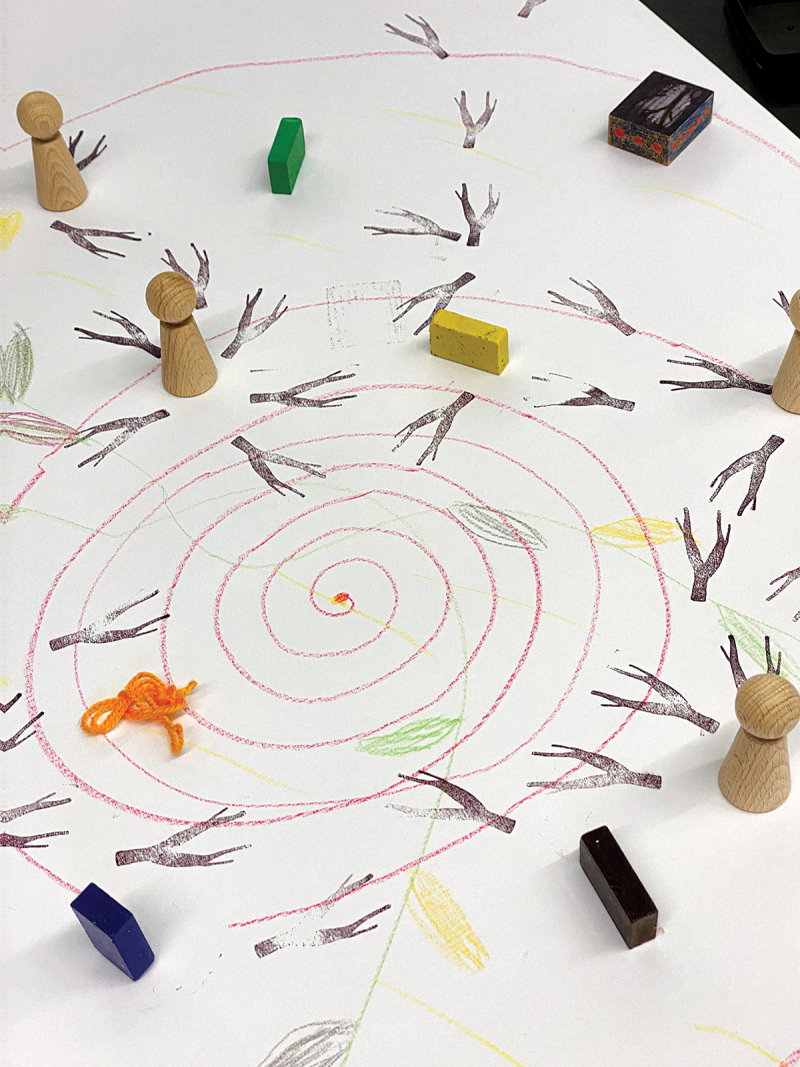
Ecosystem. Drawing made during a workshop.

This example shows how a boundary object works: people talk quickly from the object about anything and everything. The ecosystem metaphor suddenly taps into a completely different register, in this case, an existential issue about impermanence, finitude and the passing of life, which is Mode 3 knowledge. The very same metaphor also unmasked the areas where the energy stopped flowing, as when we looked at how project management and financial support were organized (See Figure 10).

Suddenly I understand why the evaluation procedures of [the funding agency] feel like a mismatch to the way our network develops. A tree doesn’t grow in a spot where it is asked to grow. It needs a place with air, other plants, roots and connecting fungi beneath the ground. I can’t predict the outcome of all the energy we invest in our network. Some initiatives will flourish, others don’t. A more dynamic and organic way of evaluation would be more supportive.

Again, the metaphor was helpful in bridging boundaries between people involved in the local networks and other stakeholders, like officers of the funding agency. A careful – because vulnerable – dialogue unfolded between project leaders and people of the funding agency, about their ‘traditional’ roles and necessary steps to evolve towards being part of a bigger ecosystem together. In this way the participatory knowledge infrastructure emerged while we were learning, connecting, and reflecting together.

## Discussion

Our study aimed to develop a new research design called dynamic knowledge synthesis. In this article we identified the characteristics and working principles of this research design, as based in a horizontal, emergent and plural epistemology to mobilize knowledge from local settings and learn together. Four working principles were key elements: (1) a rhizomatic design based on multiplicity, heterogeneity and non-linearity of knowledge; (2) fertile soil nurtured by the talents and wisdom of participants through participatory arts-based methods with invitations to potential knowledge and not-knowing; (3) a new kind of researcher (forester) with wicked skills to facilitate a learning process; and (4) the use of a metaphor as a boundary object that connects on multiple levels, for example a natural metaphor like the forest ecosystem.

In our approach to dynamic knowledge synthesis, a horizontal epistemology emerged that values scientific knowledge for objectification (Mode 1 knowledge) but equally values practical-professional knowledge developed by practitioners to solve problems (Mode 2 knowledge), as well as knowledge relating to existential issues as well as moral issues (Mode 3 knowledge) (Abma 2020a, 2020b; Kunneman 2013, 2017). This last mode of knowledge (Mode 3) is easily overlooked in our society where the seductive power of modern technology is so dominant, especially in its promise to fix all problems in the domain of health and personal well-being (Abma 2022a; Kunneman 2017). However, not all problems can be solved with Mode 1 and Mode 2 knowledge. There are matters in our lives and within professional practice that are indifferent to professional expertise and technical means, and unsolvable. Even if we can solve these issues, an ‘existential residue’ confronts us with the limits of fast, technical solutions (Abma 2022a). Schon (1987) has characterized these situations as ‘swampy lowlands’ in contrast to the high grounds of professional practice where professionals effectively use methods and techniques. Although we turn away from these situations, these are the places where ‘the greatest human concerns’ are found – and existential knowledge unfolds. Therefore, there is a need in our society for a diversity of collaborative structures to create spaces where Mode 3 knowledge can be developed. In our approach to dynamic knowledge synthesis, Mode 3 knowledge emerged as a result of co-creation. This knowledge highlighted the moral quality of the relationship between the people involved, including respect for the capacities and uniqueness of individuals, trust and sincere emotional involvement. Mode 3 knowledge often took the form of context-bound narratives and artistic-creative and embodied expressions, including the unsayable (Abma 2022b; Visse, Hansen, and Leget 2019).

We started this endeavour with the goal of making a difference and fostering mutual learning processes among all those involved in the 110 networks working towards an age-friendly world. In retrospect, we are satisfied with the positive feedback we received from many of the people active in these networks; many felt supported, energized and inspired. In terms of catalytic and empathic validity we were successful: people felt seen and understood, but they also felt empowered to act. The knowledge generated was meaningful for them and relevant for practice. This is not to say that we were satisfied with all that we had realized. We did indeed succeed in engaging older citizens, but most of them were more educated, white, physically able and eager to express themselves in group settings. We wondered why we did not hear the voices of the quiet or physically not-so-able older persons. Were more frail older people not represented and involved in the local networks, or did our methodology and approach fail to create enough space for them to feel safe to join and express themselves? We also did not succeed in reaching network members who were struggling so much that they were not able to show up at our meetings, who did not have the time or did not dare to join for other reasons. These are disturbing ethical questions related to the hopes and expectations we listed under the ethical principles including the social inclusion, equity and fairness of the process (Banks and Brydon-Miller 2018). An in-depth intersectional analysis might shine light on issues related to diversity, equity and inclusion and the colour blindness of a group of highly privileged white scholars (Muntinga et al. 2024).

This study was guided by the wish to organize a knowledge synthesis with a dynamic character. Instead of depositing knowledge in a static knowledge bank or repository, we aimed to mobilize and nourish the flowing and growing of knowledge through networks of people, so that the knowledge produced was relevant in and for daily practices. This created new challenges, such as how to report on the knowledge generated without reducing it to a few findings. We also struggled with the representation of Mode 3 knowledge and the unsayable. The rhizomatic structure helped us to envision and develop a dynamic knowledge synthesis wherein all the findings, including the existential issues as well as moral issues, were part of the horizontally connected system. Instead of a vertical, tree-like knowledge model with one overarching framework, we used and plugged in various theoretical concepts relevant for practice. We took inspiration from the work of the American theorists Jackson and Mazzei (2013), who also built on Deleuze and Guattari’s assemblage metaphor in their plea for ‘thinking-with-theory’ as a way to cross and flatten the data – theory hierarchy and other binaries (subject – object; researcher – researched). In fact, in this project, we were not only thinking-with-theory but also doing-with-theory. In the rhizomatic design, we employed several theoretical concepts to advance our understanding of the data generated.

## Epilogue

**Figure 11.**
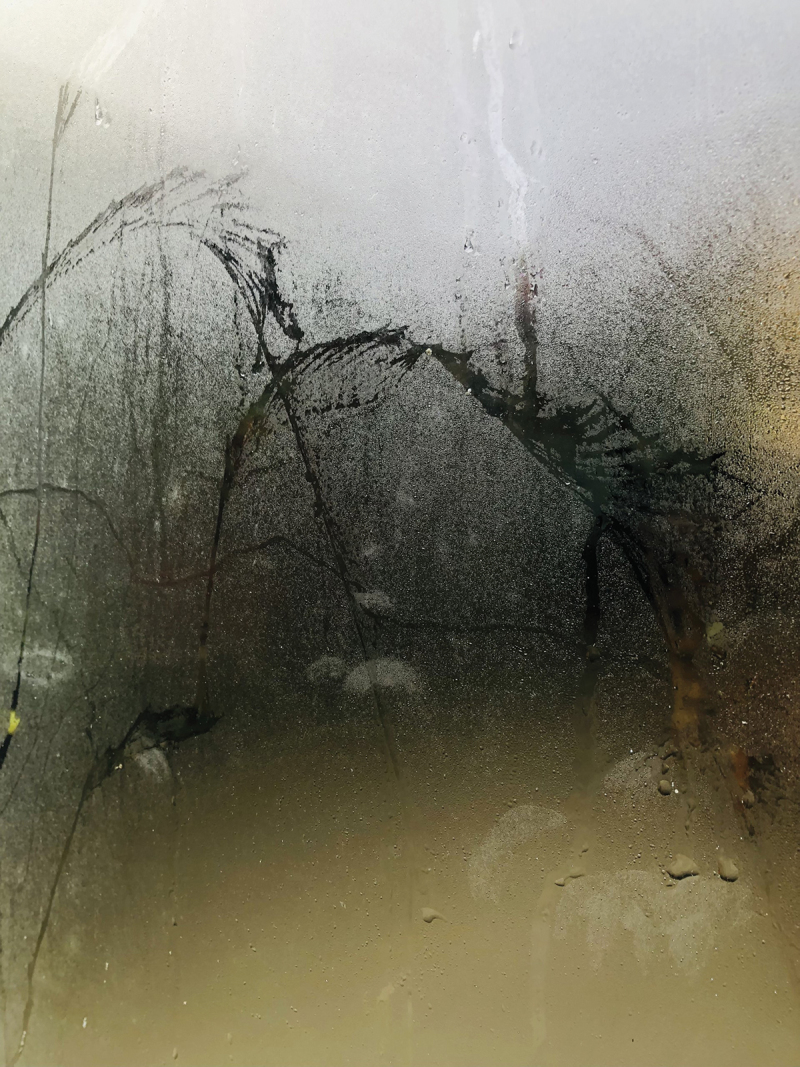
What do we see? Photograph made by the first author.

We live in a world that is becoming more complex, unpredictable, unstable, and uncertain. Crisis after crisis troubles our minds and hearts, but we still continue to rely on vertical epistemologies, although they are not really appropriate for dealing with the contemporary messiness (Abma and Groot 2023). The messier and more complex our world becomes, the more we need to mobilize and connect various pockets to unzip knowledge and hidden wisdom. This rhizomatic article is an attempt to show how we can interrupt hierarchic relationships, absolutist claims and vertical epistemologies. Dynamic knowledge synthesis is a new way to learn to embrace the complexity of ourselves and our world, inviting all the voices, pearls of wisdom, perspectives and values needed to build and envision an inclusive, (more than) human, and ethical future (See Figure 11).
